# A Hybrid Feature Extraction Method for Nepali COVID-19-Related Tweets Classification

**DOI:** 10.1155/2022/5681574

**Published:** 2022-03-09

**Authors:** T. B. Shahi, C. Sitaula, N. Paudel

**Affiliations:** ^1^Central Department of Computer Science and Information Technology, Tribhuvan University, 44600 Kathmandu, Nepal; ^2^School of Engineering and Technology, Central Queensland University, Rockhampton 4701, QLD, Australia; ^3^Department of Electrical and Computer Systems Engineering, Monash University, Clayton 3800, VIC, Australia

## Abstract

COVID-19 is one of the deadliest viruses, which has killed millions of people around the world to this date. The reason for peoples' death is not only linked to its infection but also to peoples' mental states and sentiments triggered by the fear of the virus. People's sentiments, which are predominantly available in the form of posts/tweets on social media, can be interpreted using two kinds of information: syntactical and semantic. Herein, we propose to analyze peoples' sentiment using both kinds of information (syntactical and semantic) on the COVID-19-related twitter dataset available in the Nepali language. For this, we, first, use two widely used text representation methods: TF-IDF and FastText and then combine them to achieve the hybrid features to capture the highly discriminating features. Second, we implement nine widely used machine learning classifiers (Logistic Regression, Support Vector Machine, Naive Bayes, K-Nearest Neighbor, Decision Trees, Random Forest, Extreme Tree classifier, AdaBoost, and Multilayer Perceptron), based on the three feature representation methods: TF-IDF, FastText, and Hybrid. To evaluate our methods, we use a publicly available Nepali-COVID-19 tweets dataset, NepCov19Tweets, which consists of Nepali tweets categorized into three classes (Positive, Negative, and Neutral). The evaluation results on the NepCOV19Tweets show that the hybrid feature extraction method not only outperforms the other two individual feature extraction methods while using nine different machine learning algorithms but also provides excellent performance when compared with the state-of-the-art methods.

## 1. Introduction

Natural language processing (NLP) techniques have been developed to assess peoples' sentiments on various topics. Basically, the sentiment assessment of documents into Negative, Positive, or Neutral is known as sentiment analysis. For the sentiment analysis of documents, we basically deal with sentiment classification, topic modeling, and opinion mining. Particularly, we obtain textual documents from various sources, such as social media posts and news documents. These documents reflect the peoples' feelings, whereby we would be able to identify their sentiments using machine learning techniques.

Currently, the growth of social media posts, particularly tweets, because of COVID-19, is incredibly increasing. This lets us understand people's mental stress if we process and analyze them. To this end, the design and development of an automated AI tool is essential to understand and deal with peoples' mental stresses. There are few research works of AI model developed on Nepali COVID-19-related sentiment analysis in the literature; therefore, we discuss the sentiment analysis works carried out in the Nepali language as well as few other languages, such as English.

Recent works [1–8] on COVID-19 tweets sentiment analysis in English and other languages [8] underscore the efficacy of data-driven machine learning approaches, where they employed several kinds of analysis such as topic modeling, classification, and clustering. Hence, this urges the thorough comparison of machine learning methods in sentiment analysis with the better representation of tweets for sentiment classification. For this, they used popular feature extraction methods such as TF-IDF (Term Frequency-Inverse and Document Frequency) and word embedding methods such as word2vec [9], Glove [10], and FastText [11].

With such existing works, we listed three main limitations on Nepali COVID-19-related tweet representation and classification. First, most of the existing works [2, 5, 12, 13] either use the TF-IDF or word embedding for the COVID-19 tweets written in high-resource languages, such as English. Although it might be sufficient for high-resource languages as the existing models have been trained with enough corpus, it might not be the case for low-resource languages such as the Nepali language, which uses Devanagari script (Table 1) and has a limited-size corpus [15]. And this might produce an embedding vector with less discriminating information. Second, there is no study on a detailed comparison of machine learning (ML) methods for the sentiment classification on the COVID-19-related tweets dataset, particularly in the Nepali language. The comparative study of ML methods is very important to understand the efficacy of each ML method for the current study domain. Third, there is no study of the relationship between feature types (e.g., TF-IDF and embedding) and ML methods (e.g., Support Vector Machine, Naive Bayes, etc.) on COVID-19-related tweets dataset. Notably, the ML methods perform differently according to the feature types, so we need to propose an appropriate feature extraction method to attain the optimal performance of the ML methods.

To deal with the aforementioned limitations, we, first, propose to use two pieces of information, both syntactic and semantic, called hybrid features that help understand the Nepali tweets more accurately during their representation. For the syntactic information, we adopt the TF-IDF method, which analyses the keywords/tokens based on their occurrence patterns in the training documents. For the semantic information, we employ the FastText method as suggested by Sitaula et al. [8], who carried out recent work in Nepali COVID-19-related tweets sentiment classification. Second, we compare nine different ML algorithms for the COVID-19 tweets sentiment classification. This includes the algorithms from different categories such as trees, support vector machines, and neural networks. Third, we compare and evaluate the efficacy of three feature extraction methods (TF-IDF, FastText-based, and hybrid) against nine ML methods. Based on this evaluation, we are able to identify the best ML method for the hybrid features.

The main contributions of our work are as follows:

- We propose to use three different feature extraction methods—TF-IDF, FastText-based, and TF-IDF weighted FastText-based (hybrid) features—for the representation of COVID-19-related tweets written in the Nepali language. Here, the hybrid feature extraction in Nepali language is a novel work in this study.
- We evaluate the performance of each feature extraction method on nine widely used machine learning classifiers.
- We compare and validate our proposed methods against the state-of-the-art machine learning methods on NepCov19Tweets dataset. The experimental results show that our method outperforms the existing methods in terms of classification performance.

The rest of the paper is organized as follows. Section 2 surveys the related works of COVID-19-related tweet sentiment analysis. Section 3 explains our proposed method in detail. Section 4 discusses the dataset, experimental results and compares the performance of our method with the state-of-the-art (SOTA) methods. Section 5 concludes the paper with future works.

## 2. Related Works

Natural language processing (NLP) research work in Nepali language has been well progressed in recent decades [16]. Several works on fundamental NLP applications such as part of speech tagging [17], named entity recognition [18], and text classification [14, 19] have been reported in the literature. However, there exists only one work by Sitaula et al. [8] for COVID-19-related tweets sentiment analysis in the Nepali language although there are several recent works conducted in other languages, such as English. Therefore, we review the overall research works carried out in sentiment classification based on COVID-19-related tweets in different languages, including both Nepali and non-Nepali languages.

Initially, researchers [2, 3] conducted a topic modeling for COVID-19 tweets using a Latent Dirichlet Analysis (LDA). For example, the authors in [2] inferred 26 topics initially and grouped them into ten border themes such as treatment and recovery, impact on economy and market, and impact on health and governance. Their results suggest that the themes such as “growth of case” has negative sentiments, whereas the themes such as “impact on the economy and market,” “government response,” and “treatment and recovery” have positive sentiments. Recently, Rustam et al. [1] implemented five machine learning methods: Extra Tree classifier, Decision Tree, Random Forest, XGBoost classifier, and Long Short-Term Memory (LSTM) to classify the COVID-19 tweets into three sentiments: Positive, Negative, and Neutral. Two widely used text representation methods, Bag-of-Words (BoW) and Term-Frequency and Inverse Document Frequency (TF-IDF), were used in their implementation. Their method provides the highest accuracy of 93.00% with the Extra Tree classifier (ETC). In the meantime, Kaur et al. [20] proposed a hybrid heterogeneous Support Vector Machine (HH-SVM) for the sentiment classification using COVID-19-related tweets, which show that the HH-SVM outperforms the Recurrent Neural Network (RNN).

Furthermore, the authors in [4] proposed the RNN model to classify COVID-19-related tweets into either Positive or Negative using a self-created tweets dataset and compared its performance with TextBlob [21], which shows that their method outperforms the TextBlob method. Moreover, Naseem et al. [5] employed various traditional machine learning classifiers such as Support Vector Machine (SVM), Naive Bayes (NB), and deep learning models, such as Bidirectional Long Short-Term Memory (Bi-LSTM). For the representation of tweets, they used various pretrained embedding vectors such as FastText [11], Global vectors (GloVe) [10], word2vec [9], and Bidirectional Encoder Representations from Transformers (BERT) [22]. Their method provides the highest accuracy of 92.90% in the fine-tuned BERT model.

Likewise, Basiri et al. [6] designed the ensemble deep learning model for several deep learning models such as Convolution Neural Network (CNN), Bidirectional Gated Recurrent Unit (BiGRU), and traditional ML models such as SVM and NB to perform the sentiment classification on COVID-19-related tweets. Their model yields the highest accuracy of 85.80% for the sentiment classification.

Although most of the works in COVID-19-related sentiment analysis are conducted in the English language, there are few works reported in other languages too such as Arabic [7], Brazilian [23], and Nepali [8] using both traditional machine learning and deep learning approaches. Specifically, the authors in [7] represented the COVID-19 tweets in the Arabic language using unigram and bigram coupled with TF-IDF approach and classified using various machine learning algorithms such as SVM, *K*-Nearest Neighbour (KNN), and NB. Their experiment suggests that SVM produces the highest accuracy of 85% among other classifiers. Furthermore, De et al. [23] conducted the sentiment analysis on both Brazilian news articles and COVID-19-related tweets, which suggest both news and tweets provide similar kinds of sentiments. Recently, Sitaula et al. [8] proposed the sentiment analysis of Nepali COVID-19 tweets using deep learning-based methods. They also published a benchmark dataset of COVID-19 tweets in the Nepali language, called, NepCov19Tweets dataset. They devised and trained different three convolution neural networks (CNNs) models to implement three different kinds of text representations such as FastText (fs), domain-specific (ds), and domain-agnostic (da). Finally, they combined them to build an ensemble CNN for tweet sentiment classification. Their model imparts the classification accuracy of 68.7% during sentiment classification on NepCOV19Tweets dataset. Nevertheless, their method has two limitations: first, their CNN models are complex, which could need a high computational resource for the implementation; second, given that their methods are based on only semantic features, they might be unable to capture the syntactic information. The summary of recent works on sentiment classification using COVID-19-related tweets is reported in Table 2.

Apart from the aforementioned works, there are no well-established Nepali COVID-19 tweets classification works available in the literature. However, there are few works carried out on Nepali language processing tasks closely related to sentiment classification such as Nepali text/news document classification.

Initially, Shahi and Pant [25] proposed to use the TF-IDF approach to represent the news document and classified using SVM classifier, which reports an accuracy of 74.65%. However, their method has two main limitations: first, they evaluated their methods on small-size datasets, which might require extensive work in large-size datasets for its validity; second, their method captures the syntactical information only, which means it ignores the semantic or contextual information that is important to distinguish the complex documents/tweets (e.g., higher interclass similarity and intraclass dissimilarity). Similarly, Basnet and Timalsina [26] proposed a Long Short-Term Memory (LSTM) model for the Nepali document classification, which provides an accuracy of 84.63%. Their method imparts a higher accuracy compared to Shahi and Pant [25]. However, their method is prone to overfitting problems owing to an insufficient amount of datasets. Recently, Sitaula et al. [14] devised a supervised codebook-based method for the representation of Nepali news during classification. Their method imparts the highest accuracy of 89.58% with the SVM classifier. Despite having a great promise in their method for the Nepali document representation and classification, their method still suffers from the computational burden triggered by the supervised codebook step.

## 3. Proposed Approach

**Figure 1:**
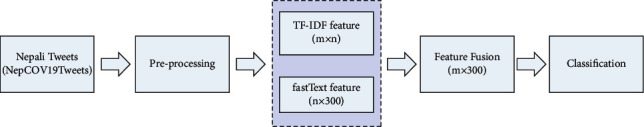
A high-level processing pipeline of our work. Note that *m* and *n* represent the number of tweets and number of tokens, respectively.

Our proposed approach consists of five steps for Nepali COVID-19 tweets sentiment classification, namely, “preprocessing”, “TF-IDF feature extraction,” “word embedding feature extraction,” “hybrid feature extraction,” and “classification.” The high-level workflow of our proposal is presented in Figure 1.

### 3.1. Preprocessing

Since preprocessing is an important step to remove noise or unnecessary tokens from the text datasets [27], we preprocess each tweet post in the dataset to sanitize the tokens for further processing. For this, we first tokenize and eliminate the alphanumeric characters. Next, we apply a rule-based approach to remove the stop words present in each tweet [16]. Last, using the stemmer algorithm, we achieve the root word of each token present in each tweet. Overall, preprocessing steps are similar to what existing researchers did in Nepali NLP processing [14].

### 3.2. TF-IDF Feature Extraction

We use a simple, yet powerful bag of word (BoW) representation method to convert each tweet into the corresponding feature vector. The BoW representation consists of three steps: tokenization, counting, and normalizing. First, we tokenize each word in a given tweet. Second, we weight each tokenized word using the term frequency-inverse document frequency (TF-IDF) as defined in the following equation:

(1) $$\begin{array}{c} \text{TF}-\text{IDF}\left(t,d\right)=\text{TF}\left(t,d\right)\times \text{IDF}\left(t\right), \end{array}$$

where TF(*t*, *d*) is the term frequency of token (*t*) in document (*d*) and IDF is defined as

(2) $$\begin{array}{c} \text{IDF}\left(t\right)=\log\frac{1+n}{1+\text{DF}\left(t\right)}+1, \end{array}$$

where DF(*t*) represents the number of tweets containing the term *t* on the dataset. Last, the TF-IDF vector (*V*) for each tweet document is normalized using L2-norm as defined in the following equation:

(3) $$\begin{array}{c} V=\frac{V}{\sqrt{V_{1}^{2}+V_{2}^{2}+\cdots V_{n}^{2}}}. \end{array}$$

### 3.3. Word Embedding Feature Extraction

Word embedding is a technique of representing a word into a fixed-size vector with the help of contextual information. They preserve the contextual information of each token, unlike the TF-IDF-based method that is purely based on the frequency of words rather than their contexts. The widely used word embedding vectors for English languages are word2vec [9], GloVe [10], and FastText [11]. Herein, we choose FastText-based word embedding in our work because it is an open-source deep learning model pretrained on large Wikipedia corpus on Nepali language and a recent study on NepCov19Tweets dataset shows that FastText-based feature extraction method is promising for the classification of Nepali COVID-19-related tweets [8]. It produces the vector of size 300-D for each word/token. As a result, a matrix of size *n* × 300 is obtained for each tweet, where *n* is the total number of tokens present in each input tweet.

(4) $$\begin{array}{c} W=\text{fastText}\left(d\right), \end{array}$$

where *W* denotes the word embedding matrix (*n* × 300) obtained from FastText-based embedding (fs) for tweet dataset *d*.

### 3.4. Feature Fusion

Similar to the role of content and context features in scene image representation more accurately as in [28], the role of syntactic and contextual information is also complementary to each other to represent the tweets more accurately. The TF-IDF method captures the syntactic information of tokens, whereas the FastText-based method captures the contextual information. Given the efficacy of both kinds of information to better represent each tweet, we propose to combine them as suggested by the authors in [29,30], for the performance improvement as shown in (5). In addition, the feature selection would be useful to reduce the feature size and boost the classification performance as suggested in [31]. However, we did not apply it because our feature size is already small enough (300-D) to train the machine learning models.

(5) $$\begin{array}{c} H_{ij}=\sum_{k=1}^{n}V_{ik}W_{kj}, \end{array}$$

where *H*_*ij*_ is the final feature matrix, *V*_*kj*_ is TF-IDF tweet matrix (*m* × *n*), and *W*_*ik*_ is a FastText-based word embedding matrix (*n* × 300). Note that *m* and *n* represent the number of tweets and number of tokens, respectively. The computational complexity of our hybrid feature is mainly based on feature fusion procedure, which is determined by the multiplication cost of two matrices (*V*_*kj*_ of size *m* × *n* and *W*_*ik*_ of size *n* × 300). Hence, the total time complexity for feature fusion is *O*(*m* × *n* × 300).

### 3.5. Classification

For the classification, we choose nine widely used machine learning classifiers: Logistic Regression (LR), Random Forest (RF), Naive Bayes (NB), K-Nearest Neighbour (KNN), Decision Tree (DT), Extra Tree Classifier (ETC), Adaptive Boosting (AdaBoost), Multilayer Perceptron-Neural network (MLP-NN), and Support Vector Machine (SVM). The selection of classifiers in this study is made based on their abilities to impart the promising classification accuracy of both Nepali and non-Nepali document analysis [1,7,25] in the literature. The short description of each classifier is presented in the following paragraphs.

#### 3.5.1. Logistic Regression (LR)

A Logistic Regression is a linear model based on the extension of linear regression analysis. In logistic regression, the range of the target variable is squeezed between 0 and 1 using

(6) $$\begin{array}{c} f\left(y\right)=\frac{1}{1+e^{-y}}, \end{array}$$

where *y* denotes the input vector.

#### 3.5.2. K-Nearest Neighbor (KNN)

K-Nearest Neighbor (KNN) is a nonparametric learning algorithm, which calculates the distance between predefined training samples and the new samples to predict the label for the new sample. Hence, it is also known as a lazy learner as it simply remembers all training samples and is nongeneralized in nature. The Euclidean, Manhattan, and Minkowski distance are the common distance functions used in K-NN classifier. In this work, we use Euclidean distance as defined in

(7) $$\begin{array}{c} d_{e}=\sqrt{\sum_{i=1}^{k}{\left(x_{i}-y_{i}\right)}^{2}}. \end{array}$$

#### 3.5.3. Naive Bayes (NB)

A Naive Bayes classifier assumes the strong independence between the pairs of input features while estimating learning parameters based on Bayes theorem of probability [32]. For the input feature vector *v*=(*v*_1_,…, *v*_*n*_), given the class *c*, the estimation of probability distribution *P*(*v*_*i*_/*c*) in (8) defines the various types of Naive Bayes classifiers. For instance, Gaussian Naive Bayes estimates the distribution parameters using the maximum likelihood function. In this work, we use Gaussian Naive Bayes implemented in Scikit-learn [33].

(8) $$\begin{array}{c} P\left(c|v_{1},v_{2}\ldots v_{n}\right)=\frac{P\left(c\right)P\left(v_{1},v_{2}\ldots v_{n}|c\right)}{p\left(v_{1},v_{2}\ldots v_{n}\right)}. \end{array}$$

#### 3.5.4. Decision Tree (DT)

Decision Tree learns the simple decision rules from the training data in a hierarchical fashion [33]. A tree is formed by recursively partitioning the dataset until it reaches the leaf node. An information criterion such as Gini index or entropy [34] is used for such partitions. In this work, classification and regression tree (CART) with Gini index is used [33] in this study.

#### 3.5.5. Random Forest (RF)

Random Forest is an ensemble of decision trees with bagging approaches [35]. It creates a forest of decision trees with random subsets of training data for each tree. The size of the subset is always the same, but the samples in the subset are drawn with replacement. Once the trees are fully formed, each test sample is travelled through each tree from root to leaf node and its label is determined from each tree. Finally, the output of all trees is averaged to get the final output of data point [36].

#### 3.5.6. Extra Tree Classifiers (ETC)

Extra Tree Classifiers (ETC) is also an ensemble learning model (similar to random forest), which constructs several randomized decision trees as week learners on various samples of training datasets and boosts the prediction accuracy. However, it is different from RF classifier in the way that trees are constructed. In ETC, further randomness is introduced, where thresholds are drawn at random for each candidate feature and the best threshold among these randomly generated thresholds is chosen as splitting rule [24] while constructing decision trees.

#### 3.5.7. AdaBoost

AdaBoost is a meta-estimator based on the adaptive boosting method of ensemble learning, which fits a sequence of weak learning trees such as small decision trees on a modified version of dataset. A strong learner is obtained by combining all such weak learners using a weighted majority voting in each boosting iteration. The data modification at each boosting iteration consists of applying weights to each of the training samples. Initially, the weights are assigned the same for all instances. Then, in each successive iteration, the weights of wrongly classified training samples are increased and as a result, it decreases the weights of training samples that were correctly classified in the previous step [37].

#### 3.5.8. Multilayer Perception Artificial Neural Network (MLP-ANN)

A Multilayer Perceptron Artificial Neural Network (MLP-ANN) is an artificial neural network algorithm of highly interconnected neurons arranged in layered fashions. It generally consists of three kinds of layers: an input layer, one or more hidden layers, and an output layer. Each neuron takes a weighted input and produces an output with an activation function. During the training operation, these weights are optimized using various optimization algorithms, such as “SGD” and “Adam.” A simple Multilayer Perception (MLP) model with one hidden layer is defined as follows.

(9) $$\begin{array}{c} \text{Output}\left(x\right)=f\left(b^{\left(2\right)}+W^{\left(2\right)}g\left(\left(b^{\left(1\right)}+W^{\left(1\right)}x\right)\right)\right), \end{array}$$

where *f* and *g* are activation functions; *b*^(1)^ and *b*^(2)^ are bias; and *W*^(1)^ and *W*^(2)^ are weight vectors. In this study, we use one hidden layer MLP with Rectified Linear Unit (ReLU) as activation function and Adam as an optimizer.

#### 3.5.9. Support Vector Machine (SVM)

The support vector machine (SVM), which optimizes a hyperplane defined in equation (10), is a binary classifier [38] in its basic form.

(10) $$\begin{array}{c} w.x-b=0, \end{array}$$

where *x*, *w*, and *b* represent feature vector, weight vector, and a bias, respectively.

The SVM uses kernel trick when the data are not linearly separable. The kernel trick implicitly maps the input feature into another feature space of higher dimension, where the data eventually become linearly separable. In this work, we use two most successful SVM kernels: Linear and Radial Bias Function (RBF) as defined in (11) and (12) and implemented in Scikit-learn [33], respectively.

(11) $$\begin{array}{c} K\left(x_{i},x_{j}\right)=\left\{x_{i}\cdot x_{j}\right\}, \end{array}$$

(12) $$\begin{array}{c} K\left(x_{i},x_{j}\right)=\exp\left(-\gamma {\left\|x_{i}-x_{j}\right\|}^{2}\right), \end{array}$$

where *K*(*x*_*i*_, *x*_*j*_)  =  *ϕ*(*x*_*i*_).*ϕ*(*x*_*j*_). Similarly, *d* and *γ* > 0 denote degree of polynomial and free parameter, respectively.

## 4. Experiment and Analysis

### 4.1. Dataset

**Figure 2:**
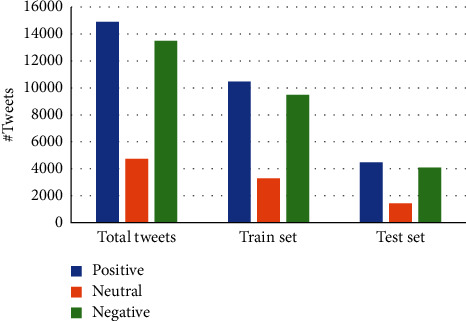
Statistical description of NepCOV19Tweets dataset [8].

We use a publicly available dataset, NepCOV19Tweets [8], for two reasons. First, data collection, annotation and preprocessing demand huge resources (human efforts and time). Second, this dataset is the most recent and only publicly available dataset related to COVID-19 in the Nepali language, which can be used to benchmark the performance of our proposed method. This dataset consists of tweets from Feb 11, 2020, to Jan 10, 2021, in the Nepali language. The detailed statistics of the dataset is presented in Figure 2.

### 4.2. Evaluation Metrics

In this section, we present the performance metrics used in our study. For the performance evaluation, we utilize commonly used metrics, such as Precision ((13), Recall ((14), *F*1-score ((15), and Accuracy ((16).

(13) $$\begin{array}{c} P=\frac{\text{TP}}{\text{TP}+\text{FP}}, \end{array}$$

(14) $$\begin{array}{c} R=\frac{\text{TP}}{\text{TP}+\text{FN}}, \end{array}$$

(15) $$\begin{array}{c} F=2\times \frac{P\times R}{P+R}, \end{array}$$

(16) $$\begin{array}{c} \text{Accuracy}\left(A\right)=\frac{\text{TP}+\text{TN}}{\text{TP}+\text{TN}+\text{FP}+\text{FN}}, \end{array}$$

where TP, TN, FP, and FN represent true positive, true negative, false positive, and false negative, respectively. Similarly, *P*, *R*, *F*, and *A* represent Precision, Recall, *F*1-score, and Accuracy, respectively.

### 4.3. Implementation

We use a popular machine learning framework, Sklearn [33], implemented in Python [39] for the implementation of the proposed methods. For the implementation on NepCOV19Tweets dataset, it is divided into train and test set in the ratio of 70 : 30 (refer to Figure 2) per category. To avoid the possible bias related to the imbalance number of samples, we report the averaged performance measures of each machine model across ten folds of the given dataset.

For the implementation of two machine learning algorithms in our study, we tune the best hyperparameters as follows: (a) ETC: \{n_estimators: (10 to 250), learning_rate: (0.2 to 1.2)\}), and (b) AdaBoost: \{n_estimators: (10 to 250), learning_rate: (0.2 to 1.2))\}. Similarly, the best hyperparameters of the remaining classifiers are chosen from previous work [8]. All the hyperparameters tuning were performed with grid search approach [40].

### 4.4. Results and Discussion

#### 4.4.1. Comparative Study of ML Classifiers on Three Different Features

Here, we compare the performance (Precision, Recall, *F*1-score, and Accuracy) of three different features type, including ours against nine machine learning classifiers. The comparative results are presented in Table 3.

**Figure 3:**
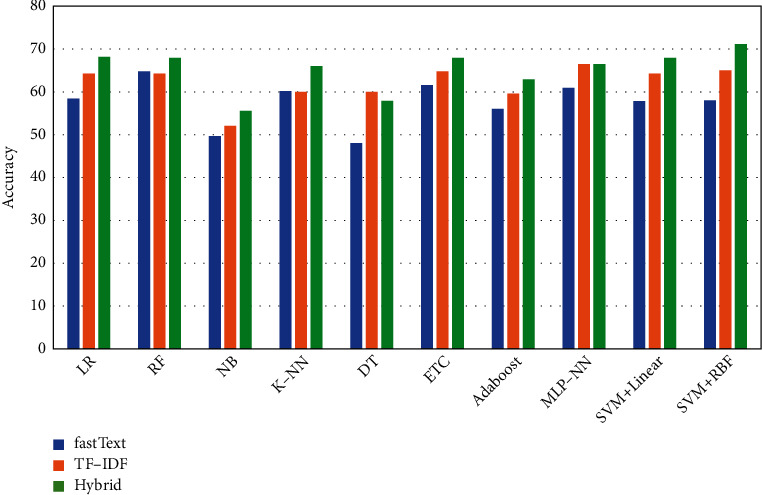
Sentiment classification accuracy of nine machine learning models with FastText, TF-IDF, and Hybrid features.

While comparing the performance of machine learning classifiers on three different feature types (TF-IDF, FastText, and hybrid), the performance varies from one machine learning algorithm to another. For the FastText-based method, we adopt a similar approach, which is the average pooling of the document matrix achieved from FastText embeddings, as suggested by Sitaula et al. [8]. Under precision metrics, K-NN, NB, and SVM + RBF have the highest performance on FastText (65.2%), TF-IDF (65.1%), and Hybrid ((71.4%), respectively. Similarly, under recall metrics, RF has the highest performance on FastText (65.4%), whereas SVM + RBF imparts the highest performance on both TF-IDF (66.0%) and Hybrid (72.1%). Furthermore, under *F*1-score metrics, K-NN, ETC, and SVM + RBF have the highest performance on FastText (65.2%), TF-IDF (62.7%), and Hybrid (70.1%) features, respectively. While comparing the ML methods in terms of classification accuracy, we observe that RF produces the highest performance on FastText (64.8%), and SVM + RBF imparts the highest accuracy on both TF-IDF (65.1%) and Hybrid (72.1%) features. From the above analysis, we stipulate that SVM + RBF is the best performing method on hybrid (proposed) features and TF-IDF features, whereas RF possesses an ability to outperform other methods on FastText method. Overall, we observe that most of the classifiers improve their performance (Precision, Recall, and *F*1-score) on hybrid features (Figure 3).

We notice that hybrid features for sentiment classification outperform the other two feature extraction methods (FastText and TF-IDF) in terms of Precision, Recall, *F*1-score, and Accuracy in most of the machine learning (ML) classifiers. For example, the SVM + RBF kernel imparts the highest performance of 71.4%, 72.1%, 70.1%, and 72.1% for Precision, Recall, *F*1-score, and Accuracy, respectively, when using the hybrid features. Similarly, TF-IDF is the second-best performing feature, which imparts a higher performance than the FastText-based method on most of the ML algorithms. As an example, SVM + RBF imparts Precision of 66.0%, Recall of 66.0%, *F*1-score of 62.1%, and an Accuracy of 65.1% on TF-IDF features. In contrast, the FastText-based features are the least-performing method, which has the lowest performance in most of the cases against two other counterparts. To this end, such encouraging results suggest that hybrid features have more discriminating information compared to other counterparts during classification.

#### 4.4.2. Class-Wise Study of Classifiers' Performance on Hybrid Features

To understand the performance of the hybrid features on a deeper level, we perform the class-wise performance analysis of each machine learning classifier. The evaluation results are presented in Table 4.

**Figure 4:**
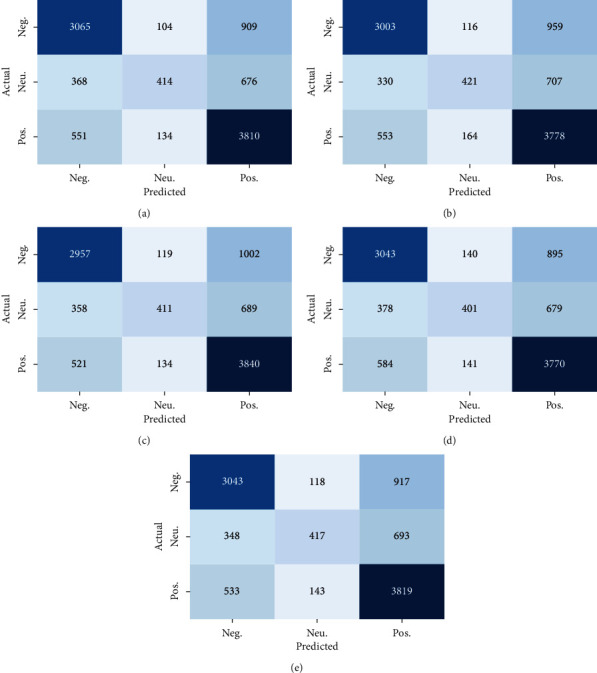
Confusion matrix produced by the best performing ML method (SVM + RBF) on NepCOV19Tweets (5 sets) using hybrid features. Note that Pos., Neu., and Neg. denote Positive, Neutral, and Negative classes, respectively.

**Figure 5:**
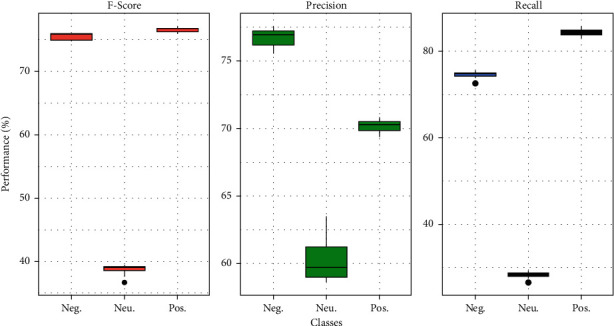
Class-wise box-plot analysis of performance metrics over results of ten folds used in this study for the best performing method (SVM + RBF) on hybrid features. Note that Neg., Neu., and Pos. represent Negative, Neutral, and Positive classes, respectively.

While looking at the performance of each ML classifier in the Positive class, we notice that SVM + RBF provides the highest Precision (69.7%), ETC provides the highest Recall (87.7%), and SVM + RBF provides the highest *F*1-score (75.9%). Similarly, ETC imparts the highest Precision of 73.8%, NB produces the highest Recall of 51.6%, and *F*1-score of 33.6% for the Neutral class. Furthermore, SVM + RBF imparts the highest Precision (74.4%), Recall (76.9%), and *F*1-score (75.6%) for the Negative class. To this end, we believe that SVM + RBF on hybrid features produces an encouraging performance in class-wise measurement as well. In addition, we observe the class-wise results produced by our method using confusion matrix (Figure 4) and box-plots analysis (Figure 5), which show that the Neutral class is more challenging than the Positive and Negative classes. This is because the Neutral class contains both positive and negative information. As a result, most of the classifiers, including SVM + RBF, are misclassifying tweets belonging to this category. From the box-plots analysis (Figure 5), we also notice that our method shows the stable and robust performance across all three classes during classification.

#### 4.4.3. Comparison of Our Method with the State-of-the-Art Methods

We also compare our method with the existing state-of-the-art methods, which are presented in Table 5.

While looking at Table 5, we notice that our method produces the highest classification accuracy of 72.1% on NepCov19Tweets dataset, which is at least 10.0% higher than the least-performing method (Shahi and Pant [25]) and over 3.4% higher than the second-best method (Sitaula et al. [8]). In addition, our method achieves a lower feature size (300-D) compared to the second-best method (320-D). Such significant improvement in averaged classification performance along with the lower feature size is our main achievement in this study.

In addition, the feature extraction methods adopted in the previous methods basically rely on syntactic information only (except Sitaula et al. [8]), but the textual documents also require semantic information for better discriminability. As such, our method is able to attain the prominent classification performance by the help of both kinds of information (syntactic and semantic) altogether. Thus, we believe that it is very important to consider both kinds of information for the feature extraction during classification process.

## 5. Conclusion and Future Works

In this paper, we have proposed to use hybrid features (FastText + TF-IDF) to represent Nepali COVID-19-related tweets for the sentiment classification. Also, we have evaluated the classification performance of nine machine learning algorithms over the proposed hybrid features. The experimental results reveal that the proposed hybrid features outperform each individual (FastText and TF-IDF) feature during the sentiment classification. The SVM + RBF is the best performing classifier with overall 72.1% classification accuracy. The class-wise investigation on NepCov19Tweets dataset divulges that “Neutral” class is the most challenging to classify than other two (Positive and Negative) classes for most of the learning classifiers. Moreover, the comparison of our method with the state-of-the-art methods accentuate that our method imparts significantly better classification performance.

In contrast, our method has two major limitations. First, our method has only one kind of contextual (semantic) information achieved from FastText. Thus, the addition of multiple contextual information achieved from other models such as Glove and Word2Vec might help improve the performance. Second, our method uses traditional ML methods for the evaluation, which is not end to end. Thus, the development of end-to-end deep learning model using such approach might be useful in the future to learn more interesting spatial and temporal information for the sentiment classification.

## Figures and Tables

**Table 1:** Alphabets and numerals used in the Nepali language [14].

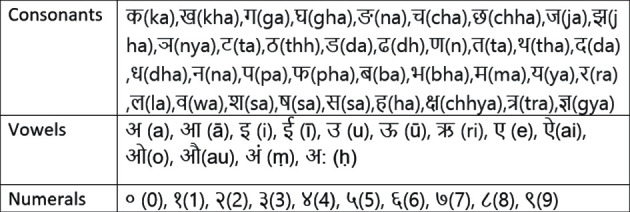

**Table 2:** Summary of some recent works on sentiment classification using COVID-19-related tweets.

Methods	Dataset	Accuracy	Reference
Ensemble CNN	NepCov19Tweets	68.70	[8]
Extra tree classifier	Covid-19Tweets	93.00	[24]
Fusion-based DL	StandfordSentiment40	85.80	[6]
SVM	Self-created dataset	85.00	[7]
H-SVM	Self-created dataset	96.30	[20]
Naseem et al. [5]	COVIDSenti	92.90	[5]

**Table 3:** Comparison of performance of three feature extraction methods with nine machine learning algorithms in terms of classification performance (%).

Classifiers	FastText				TF-IDF				Hybrid			
	*P*	*R*	*F*	*A*	*P*	*R*	*F*	*A*	*P*	*R*	*F*	*A*
LR	58.4	58.5	54.4	58.5	62.9	64.4	60.7	64.4	65.9	68.1	65.9	68.1
K-NN	**6**5.2	65.2	**6**5.2	60.2	58.0	61.0	58.0	60.1	64.1	66.0	63.6	66.0
NB	54.1	50.0	50.1	49.6	**6**5.1	52.1	55.0	52.0	62.4	55.5	57.7	55.6
DT	48.4	48.0	48.2	48.0	58.1	60.5	58.6	59.5	57.0	57.9	57.4	57.9
RF	63.1	**6**5.4	62.5	**6**4.8	63.1	65.1	62.0	64.0	68.7	67.9	64.6	67.9
ETC	63.5	61.6	58.4	61.6	63.1	64.9	**6**2.7	64.9	69.2	67.9	64.8	67.9
AdaBoost	54.2	56.1	52.9	56.1	63.7	62.7	58.2	59.6	60.1	62.9	60.1	62.9
MLP-NN	61.3	60.9	56.5	60.9	58.6	60.6	59.2	60.6	66.5	66.4	66.4	66.4
SVM + Linear	51.2	58.3	52.2	57.8	62.1	64.3	59.2	64.2	66.0	68.0	64.6	68.0
**SVM** + RBF	62.1	58.1	53.0	58.0	66.0	**6**6.0	62.1	**65**.1	**7**1.4	**7**2.1	**7**0.1	**7**2.1

Note that *P*, *R*, *F*, and *A* denote overall Precision, Recall, *F*1-score, and Accuracy for three types of feature extraction methods (FastText, TF-IDF, and Hybrid), respectively. The best hyperparameters of each machine learning algorithm are as follows: LR (C:10, solver: lbfgs, and max_iteration: 2000), K-NN (leaf_size: 35, n_neighbour: 120, p: 1), DT (criterion: gini, min_sample_leaf: 10, and min_sample_split: 2), RF (min_sample_split: 6, min_sample_leaf: 3), ETC (min_sample_leaf: 1, min_sample_split: 2, and n_estimator: 200), AdaBoost (learning_rate: 0.8, n_estimator: 100), MLP-NN (hidden_layer_size: 20, learning_rate_init: 0.01, solver: Adam, and max_iteration: 2000), SVM + Linear (c: 1, Gamma: 0.1), and SVM + RBF (c: 100, Gamma: 0.1). The highest metrics are highlighted in boldface.

**Table 4:** Class-wise performance of proposed hybrid features with nine machine learning algorithms (LR, KNN, NB, DT, RF, ETC, AdaBoost, MLP-NN, and SVM).

Classifiers	Positive			Neutral			Negative		
	*P*	*R*	*F*	*P*	*R*	*F*	*P*	*R*	*F*
LR	67.9	79.4	73.2	44.1	16.6	24.1	71.4	74.0	72.7
KNN	65.4	79.0	71.5	45.7	15.1	22.7	69.2	69.9	69.9
NB	66.8	56.9	61.4	24.9	**5**1.6	**3**3.6	71.0	55.4	62.3
DT	62.5	63.6	63.0	26.4	21.9	23.9	61.9	64.4	63.1
RF	64.6	84.7	73.3	70.0	10.7	18.6	72.8	69.9	71.3
ETC	63.5	**8**7.7	73.7	**7**3.8	12.2	20.9	75.9	66.6	71.0
AdaBoost	64.2	79.4	71.0	46.3	11.2	18.1	68.4	69.2	68.8
MLP-NN	69.7	76.2	72.8	40.	25.9	31.7	71.	73.6	72.9
SVM + Linear	67.1	80.7	73.3	50.4	09.0	15.3	70.2	75.1	72.6
SVM + RBF	**6**9.7	83.4	**7**5.9	58.7	17.9	27.4	**7**4.4	**7**6.9	**7**5.6

Note that *P*, *R*, and *F* denote Precision, Recall, and *F*1-score for three classes (Positive, Neutral, and Negative).

**Table 5:** Comparison of our method against the state-of-the-art methods using averaged classification accuracy (%) on NepCov19Tweets dataset.

Methods	Feature size	Accuracy
Shahi and Pant [25], 2018	300-D	62.1
Basnet and Timalsina [26], 2018	300-D	62.9
Sitaula et al. [14], 2021	17-D	59.8
Sitaula et al. [8](ds), 2021	3-D	61.5
Sitaula et al. [8] (da), 2021	17-D	59.5
Sitaula et al. [8] (ft), 2021	300-D	68.1
Sitaula et al. [8] (ds + da + ft), 2021	320-D	68.7
**Ours**	300-D	**72.1**

## Data Availability

The data are publicly available from the Kaggle data repository (https://www.kaggle.com/mathew11111/nepcov19tweets).
